# Age-Associated Changes In Oxidative Stress and NAD^+^ Metabolism In Human Tissue

**DOI:** 10.1371/journal.pone.0042357

**Published:** 2012-07-27

**Authors:** Hassina Massudi, Ross Grant, Nady Braidy, Jade Guest, Bruce Farnsworth, Gilles J. Guillemin

**Affiliations:** 1 Department of Pharmacology, School of Medical Sciences, Faculty of Medicine, University of New South Wales, Sydney, New South Wales, Australia; 2 Australasian Research Institute, Sydney Adventist Hospital, Sydney, New South Wales, Australia; 3 School of Psychiatry, University of New South Wales, Faculty of Medicine, Sydney, New South Wales, Australia; 4 St Vincent's Centre for Applied Medical Research, Sydney, New South Wales, Australia; Texas A&M University, United States of America

## Abstract

Nicotinamide adenine dinucleotide (NAD^+^) is an essential electron transporter in mitochondrial respiration and oxidative phosphorylation. In genomic DNA, NAD^+^ also represents the sole substrate for the nuclear repair enzyme, poly(ADP-ribose) polymerase (PARP) and the sirtuin family of NAD-dependent histone deacetylases. Age associated increases in oxidative nuclear damage have been associated with PARP-mediated NAD^+^ depletion and loss of SIRT1 activity in rodents. In this study, we further investigated whether these same associations were present in aging human tissue. Human pelvic skin samples were obtained from consenting patients aged between 15–77 and newborn babies (0–1 year old) (n = 49) previously scheduled for an unrelated surgical procedure. DNA damage correlated strongly with age in both males (p = 0.029; r = 0.490) and females (p = 0.003; r = 0.600) whereas lipid oxidation (MDA) levels increased with age in males (p = 0.004; r = 0.623) but not females (p = 0.3734; r = 0.200). PARP activity significantly increased with age in males (p<0.0001; r = 0.768) and inversely correlated with tissue NAD^+^ levels (p = 0.0003; r = −0.639). These associations were less evident in females. A strong negative correlation was observed between NAD^+^ levels and age in both males (p = 0.001; r = −0.706) and females (p = 0.01; r = −0.537). SIRT1 activity also negatively correlated with age in males (p = 0.007; r = −0.612) but not in females. Strong positive correlations were also observed between lipid peroxidation and DNA damage (p<0.0001; r = 0.4962), and PARP activity and NAD^+^ levels (p = 0.0213; r = 0.5241) in post pubescent males. This study provides quantitative evidence in support of the hypothesis that hyperactivation of PARP due to an accumulation of oxidative damage to DNA during aging may be responsible for increased NAD^+^ catabolism in human tissue. The resulting NAD^+^ depletion may play a major role in the aging process, by limiting energy production, DNA repair and genomic signalling.

## Introduction

Aging is a time-dependent progressive decline in biochemical and physiological function, associated with increased risk of mortality and morbidity [1], [2]. There is a growing awareness that oxidative stress (OS) plays a key role not only in the aging process, but also in various degenerative diseases including Alzheimer's disease, cancer, diabetes, and chronic inflammation [3]. The OS theory, first proposed by Harman (1956), suggests that age-related biochemical and physiological decline is associated with a chronic state of imbalance between the production of oxidants and the intracellular antioxidant capacity. This can trigger deleterious changes in numerous cellular processes, leading to a loss in metabolic function [4]. Reactive oxygen species (ROS), such as hydroxyl radicals (HO⋅^−^), superoxide anions (O_2_^⋅^) and hydrogen peroxide (H_2_O_2_) are continuously produced endogenously as by-products of normal cellular respiration. At low concentrations, they are important in a variety of cellular activities such as immune function and vasodilation [5]. However, at high concentrations, they are capable of damaging proteins, lipids and DNA [6].

Lipid peroxidation resulting from oxidative damage to lipids, occurs as a chain reaction where ROS attack polyunsaturated fatty acids present in the lipid membrane to produce highly reactive lipoperoxides in the form of thiobarbituric acid reactive substances (TBARS), and the reactive aldehydes such as malondialdehyde (MDA), and 4-hydroxy-2-nonenal (4-HNE), which can be used as indices of lipid peroxidation [7]. Studies showing an increase in intracellular ROS through exogenous hydrogen peroxide treatment *in vitro*, or inhibition of endogenous ROS scavenging enzymes such as superoxide dismutase (SOD) and catalase, have been shown to promote premature aging and significantly lower lifespan [8]–[10]

While there are several sources of ROS, it is generally accepted that the mitochondria (the site of oxidative phosphorylation and ATP generation) is the major source of ROS production [11]–[14]. During rapid ATP turnover, the electron transport chain (ETC) in the mitochondria are continuously involved in reducing molecular oxygen to water in a four electron reduction process. However, a small percentage of oxygen consumed escapes the ETC as O_2_^⋅−^ which can generate other endogenous ROS, therefore posing a significant threat to aerobic organisms, and their intracellular constituents [15]. Previous studies have shown that age-associated mitochondrial dysfunction increases with age. This can severely compromise the efficiency of the ETC, resulting further ROS generation. Harper et al. (1998) showed that O_2_^⋅−^ leakage was significantly higher in isolated hepatocytes cultured from 30 month old mice, compared to 3 month old mice [16]. Impairment in mitochondrial function and the consequent reduction in ATP-production may explain both the pro-oxidant shift and energy deficit in aging [17], [18].

Oxidative DNA damage is a major factor associated with age-related diseases. It can interfere with the expression of various genes associated with DNA repair and cellular proliferation [8]. The human body has a number of physiological protection and repair systems, including activation of the DNA nick sensor poly(ADP-ribose) polymerase-1 (PARP), and the natural endogenous antioxidant defense system to attenuate free radical induced damage, and thereby maintain cellular homeostasis [9], [19].

Essential to the PARP mediated repair of oxidative DNA damage is nicotinamide adenine dinucleotide (NAD^+^). This ubiquitous biomolecule is involved in several cellular redox reactions in all living organisms, and serves not only as a substrate for PARP but also for another important class of enzymes known as sirtuins or silent information regulators of gene transcription [20]. PARP activation in response to DNA strand breaks can consume large quantities of NAD^+^ to produce poly(ADP-ribose) polymers on target proteins in a process called poly-ADP-ribosylation [21]. Under conditions of mild-to-moderate DNA damage, activation of PARP-1 leads to DNA repair and restoration of normal cellular function [22]. However, excessive DNA damage leads to over-activation of PARP, and increased NAD^+^ catabolism [23], [24], resulting in suppression of NAD^+^-dependent ATP generation and possible energy crisis.

Importantly, NAD^+^ levels also regulate the activity of the sirtuin family of enzymes [25], [26]. As mentioned previously, sirtuins are a highly conserved family of nuclear regulatory proteins that use NAD^+^ as their substrate in a reaction to remove an acetyl group from the lysine residue [27]. SIRT1 (located in the nucleus), is thought to play a critical role in aging and cell longevity through its modulation of key transcription factors, such as p53. Both human and mouse SIRT1 are thought to promote cell survival by deacetylating and thus deactivating the p53 proapoptotic gene [28]–[30]. Adequate NAD^+^ levels maintain SIRT1 activity which can delay apoptosis and provide vulnerable cells with additional time to repair, even after repeated oxidative stress insult. While extensive research has examined the role of SIRT1 in aging [31]–[34], no study has investigated the impact of age on human tissue NAD^+^ levels or correlated SIRT1activity with NAD^+^ levels

As over-activation of PARP-1 following extensive DNA damage leads to a substantial increase in NAD^+^ catabolism, the decline in NAD^+^ and the rise in nicotinamide have the potential to significantly down regulate SIRT1 deacetylase activity [35]. Our laboratory recently reported a significant reduction in NAD^+^ levels and SIRT1 activity in physiologically aged female Wistar rats [36]. Based on this and previous studies by others, we hypothesize, that markers for oxidative stress will increase with age in human tissue and correlate with an increase in PARP activity, NAD^+^ depletion and reduced SIRT1 activity. The aim of this study therefore, was to quantify, markers for oxidative stress (i.e. lipid peroxidation and DNA damage), PARP and SIRT1 activities, and NAD^+^ levels in non-sun exposed human tissue from participants across a wide age range.

## Materials and Methods

### Reagents and Chemicals

Nam, NAD^+^, β-nicotinamide adenine dinucleotide reduced form (NADH), 3-[-4,5-dimethylthiazol-2-yl]-2,5-diphenyl tetrazolium bromide (MTT), alcohol dehydrogenase (ADH), bicine, TRIS, N-(1-naphthyl) ethylenediamine dihydrochloride, EGTA, EDTA, γ-globulins, dipotassium orthophosphate, bovine serum albumin (BSA), tricarboxylic acid (TCA), Triton X-100, SDS, HEPES, 1-dithio DL-threitol (DTT), malondialdehyde (MDA), and thiobarbituric acid (TBA) were obtained from Sigma-Aldrich (Castle-Hill, Australia). Phosphate buffer solution (PBS) was from Invitrogen (Melbourne, Australia). Phenazine methosulfate (PMS) was obtained from ICN Biochemicals (Ohio, USA). Bradford reagent was obtained from BioRad (Hercules, CA, USA).

### Participants

This study was conducted in accordance with the Helsinki declaration. Approval was obtained from the Sydney Adventist Hospital Ethics Committee at the Sydney Adventist Hospital (HREC#25/10). Human skin tissues were obtained with informed consent from patients (n = 49) who were scheduled for surgery at the Sydney Adventist Hospital. The participants included patients aged between 15–77 and newborns babies (0–1 year old). Patient anonymity was preserved throughout the study.

### Sample preparation

Skin tissues were obtained from non-sun exposed areas of the pelvic region. Surgically removed tissues were washed twice with PBS within two minutes of excision. They were then dissected into several equivalent pieces, and placed on dry ice for transport to the to the liquid nitrogen storage tank (<10 minutes). The skin samples were homogenized using a Labserve tissue homogenizer (model D-130) in (i) Nam homogenate solution for NAD(H) and DNA damage assays, (ii) PARP lysis buffer for PARP activity and (iii) the Tris-HCl buffer for SIRT1 activity. The homogenates were centrifuged at 12000 rpm for 10 minutes and the supernatant was extracted for analysis.

### Exclusion Criteria

A) Participants were excluded from the study if any one of the following conditions were present: (i) chronic non-localised systemic inflammatory conditions (e.g. infection), (ii) pregnant (iii) current use of chemotherapeutic drugs.

B) Patients under the age of 45 were excluded from the study if (i) they were currently taking anti-inflammatory medications such as methatrexate and cortosine, (ii) had evidence of active autoimmune disease, including arthritis, (iii) were current smokers or ex-smokers who ceased within one year, and (iv) had evidence of skin cancer.

### Measurement of Intracellular NAD^+^ levels

Intracellular NADH and total NAD(H) concentrations in tissue homogenates and RBC samples were measured spectrophotometrically using the thiazolyl blue microcycling assay established by Bernofsky and Swan (1973), and adapted for 96 well plate format by Grant and Kapoor (1998) [37], [38]. In brief, 125 µl of the reaction mixture containing 100 mM Bicine (pH 7.8), 0.42 mM MTT and 1.66 mM PMS was added to 6 µl of the sample. For Total NAD (H) measurement, 20 µl of ADH in 0.15% ethanol was added to the reaction mixture. The amount of Total NAD(H) and NADH concentrations were measured as the change in absorbance at 570 nm at 37°C for 10 minutes with a Model 680XR microplate reader (BioRad, Hercules). Finally, the amount of NAD^+^ was calculated as the difference between the Total NAD(H) and NADH concentrations.

### Measurement of Malondialdehyde-Thiobarbituric Acid (MDA) as a Marker for Lipid Peroxidation

The level of lipid peroxidation was quantified by measuring the amount of malondialdehyde-thiobarbituric acid (MDA-TBA) adduct formed by the reaction of MDA and TBA at 100°C in tissue samples. MDA levels were measured using a standardised commercial assay kit (Caymen Chemical Co. Ann Arbor, MI USA) according to the manufacturer's instructions. Briefly, 50 µl of sample was added to 50 µl SDS solution and 1 ml TBA and incubated at 100°C for 1 hr. Afterwards, the samples were placed on ice for 10 minutes to terminate the reaction, and centrifuged at 1,600 g for 10 min to remove debris. The absorbance for the newly formed product was read at 540 nm using the Model 680XR microplate reader (BioRad, Hercules, USA).

### Isolation and Extraction of Nuclei for DNA Damage Assay in tissue

Tissue homogenates were spun through 300 µl of homogenate solution containing nicotinamide in water (pH 7.4) for 10 mins. The pellet was washed three times with a cold buffer containing 100 µl of 1 M HEPES (PH 7.9), 40 µl of 2.5 M KCL, 2 µl of 0.5 M EDTA, 10 µl of 0.5 M EGTA and 1 µl of 1 M DTT in 9.848 ml of deionised water. The nuclei was later suspended in 4 ml extraction buffer containing 200 µl of 1 M HEPES (PH 7.9), 1.6 ml of 2.5 M KCL, 20 µl of 0.5 M EDTA, 100 µl of 0.5 M EGTA, 1 ml of 1 M DTT, 7.08 ml of deionised water and glycerol 100%. The samples were then allowed to shake on an orbital shaker for one hour. The final pellet was obtained after centrifugation at 13,000 g at 4°C.

### DNA Damage Assay

Phosphorylation of H2AX at serine 139 as a marker for DNA damage was used to quantify the amount of DNA damage caused in human skin tissues using an established kit (Active Motif, Carlsbad, California, USA) according to the manufacturer's instructions and as previously described [36]. In brief, 5 µg/ml of anti-phospho-H2AX-ser139 was added to the nuclear extracts in PBS and allowed to incubate overnight at 4°C. The primary antibody was removed by washing the nuclear extracts 3 times with 200 µl PBS. Alexis 488 goat anti-rabbit IgG secondary antibody (1∶1000) was added to the sample and incubated for 1 hour at room temperature. The secondary antibody was removed by washing the sample twice with 200 µl PBS. The fluorescence was read using Fluostar Optima Fluorometer (NY, USA). Filter excitation and emission was set at 485 nm and 520 wavelengths respectively.

### PARP Activity Assay

PARP activity was measured in tissue homogenates using a new operational protocol relying on the chemical quantification of NAD^+^ modified from Putt et al. (2005) and adapted for 96 well plate format by Braidy et al. (2009) [39], [40]. Briefly, 200 µl of PARP lysing buffer containing 10 mM MgCl_2_, 1% Triton X-100, and 20 µM NAD^+^ in 50 mM Tris buffer (pH 8.1) was added to the tissue homogenate. The sample was then incubated for 1 hour at 37°C and the amount of NAD^+^ consumed was measured by the NAD(H) microcycling assay using the Model 680XR microplate reader (BioRad, Hercules).

### SIRT1 deacetylase Activity

SIRT1 deacetylase activity was evaluated in tissue extracts using the SIRT1 Deacetylase Flourometric Assay Kit (Sigma-Aldrich, Castle Hill, Australia) according to the manufacturer's instructions. In brief, 10 µl of the tissue homogenate was added onto a black 96 well plate well containing 30 µl of the 50 mM Tris-HCl buffer (pH 8.8) containing 4 mM MgCl_2_, 0.5 mM DTT, 200 µM NAD^+^ and 10 µl of the fluoro substrate peptide solution. The SIRT1 activity in the samples was measured by comparing the fluorescence obtained against the SIRT1 standard curve using Fluostar Optima Fluorometer (NY, USA). Filter excitation and emission was set at 340 nm and 430 wavelengths respectively.

### Bradford Protein Assay

Intracellular NAD (H) concentrations, MDA-TBAR levels, PARP, DNA damage and SIRT1 activities were adjusted for variations in protein using the standard Bradford Protein Assay [41].

### Data Analysis

Results are presented as the mean ± the standard error of the mean (SEM) unless otherwise stated. One-way ANOVA followed by Tukey's post-hoc test were used to determine statistical significance between normally distributed groups. Kolmogorov-Smirnov test was used to test for normality. Comparison of non-normally distributed groups were carried out using the non-parametric Mann-Whitney U-Test. Correlation between variables was determined using either Pearson's or spearman's correlation for normally and non-normally distributed data respectively. Statistics were performed using GraphPad Prism and GraphPad Instat (San Diego CA). Differences between stratified groups were considered significant if *p* was less than 0.05.

## Results

### Changes in lipid peroxidation, DNA damage, NAD^+^ levels, PARP and SIRT1 activities between defined age categories

Treating the population as a whole, we observed a significant difference in MDA between the four age categories, Newborns (0–1 years), adults (30–50 years), older adult (51–70 years), elderly (>70 years)., MDA (lipid peroxidation) levels were significantly higher in elderly subjects compared to newborns and young adults (Table 1; p<0.05). DNA damage was also higher in elderly subjects compared to newborns (p<0.05), young adults (p<0.05), and adult subjects (p<0.05) (Table 1). PARP activity was significantly increased in adults, older adults and elderly subjects compared to newborns (Table 1; p 0.05). A significant decrease in total NAD^+^ content was observed in adults (p<0.05), older adult (p<0.05) and elderly (p<0.05) subjects compared to newborns (Table 1). Evaluation the sample population as a whole, we found no significant difference in SIRT1 activity between any of the four age categories (p>0.05; Table 1).

**Table 1:** Differences between discrete categorised variable of the entire sample population.

Characteristic/Test	Newborns (0–1 years)	Young Adults (30–50 years)	Middle Age (51–70 years)	Elderly (>71 years)
Number in age groups (M/F)	8 (8/0)	12 (4/8)	23 (12/11)	5 (2/3)
Average Age mean yrs ± SEM	0.072±0.00	41.17±1.88	63.17±1.31	74.4±1.08
TBARS (mM MDA/mg protein), mean ± SEM	1.56±0.19	1.72±0.37	2.46±0.32	4.21±1.36* ^, ^ **
pH2AX (FU/mg protein), mean ± SEM	12.83±2.24	21.70±4.69	46.05±1.30	123.40±1.08* ^, ^ ** ^, ^ ***
PARP Activity (ng NAD^+^ used/hr/mg protein), mean ± SEM	2.52±0.44	17.73±2.51*	20.69±1.63*	19.52±3.94*
NAD^+^ (ng NAD^+^/mg protein), mean ± SEM	8.54±1.55	2.74±0.41*	1.08±0.15*	1.06±0.19*
SIRT1 Activity (FU/mg protein), mean ± SEM	27.13±0.74	30.41±3.12	24.65±4.38	11.83±11.33

Data presented as mean ± SEM.

* p<0.05 compared to newborns,

** p<0.05 compared to young adults (30–50 years),

*** p<0.05 compared to middle age (51–70 years).

### Age- and Gender related changes in Lipid Peroxidation

**Figure 1:**
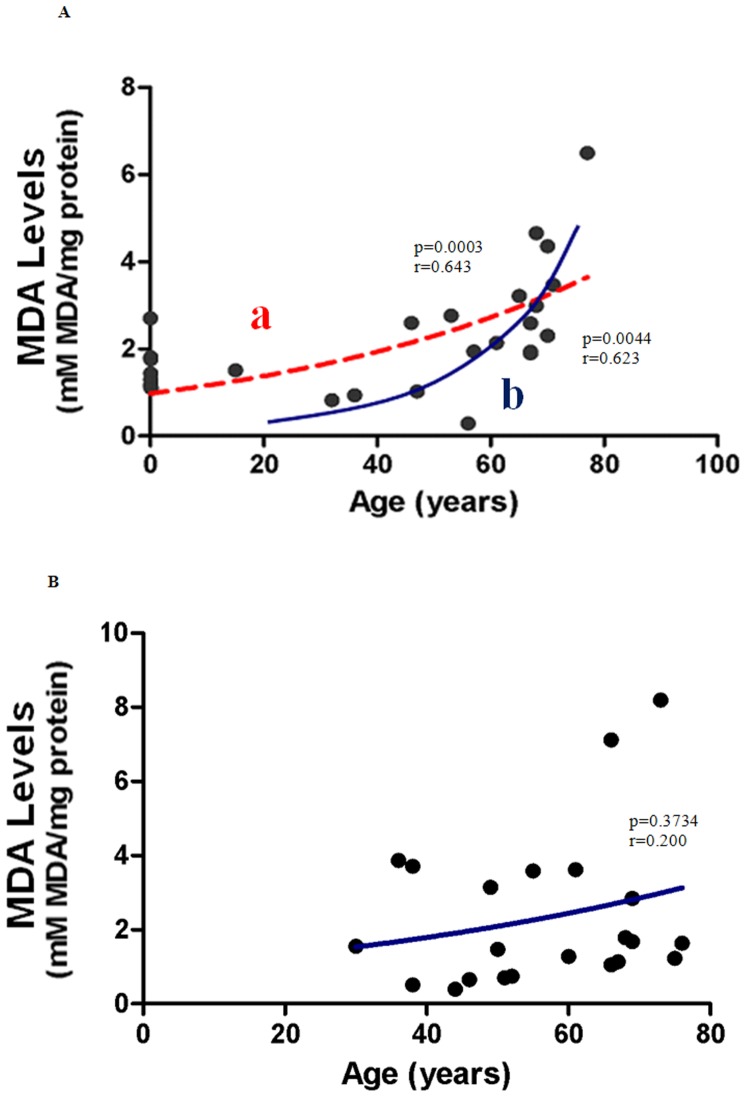
Effect of age on tissue lipid peroxidation in (A) males (B) females. (**A**) **Lipid peroxidation increases with age in male subjects.** MDA levels in human tissue increased significantly with age in males aged between 0–77 years (Line a; p = 0.0003; n = 27). Line b shows post-pubescent (males only) data (p = 0.0044; n = 19). Spearman's correlation coefficient for the non-normally distributed population was r = 0.643 and r = 0.623 for line a, and line b respectively. An exponential (first-order) least squares fit was used to generate the nonlinear trend lines (line a and b). (**B**) **Changes in lipid peroxidation with age in female subjects.** The apparent increase in MDA levels in post-pubescent females (36–76 years) is not statistically significant (p = 0.3734; n = 22). Spearman's Correlation coefficient for a non-normally distributed population was 0.200. An exponential (first-order) least squares fit was used to generate the nonlinear trend line.

Lipid peroxidation in our skin samples was quantified by measuring a primary by-product malondealdehyde (MDA). MDA levels strongly correlated with age for males aged between 0–77 years (Fig. 1A, line a; p<0.05). The effect of increasing age was more pronounced in post-pubescent males (Fig. 1A, line b; p<0.05) compared to age matched post-pubescent females (Fig. 1B; p>0.05) .The apparent increase in MDA formation with age in females did not reach statistical significance (p = 0.3734) (Fig. 1B).

### Gender-related differences in DNA Damage during Aging

**Figure 2:**
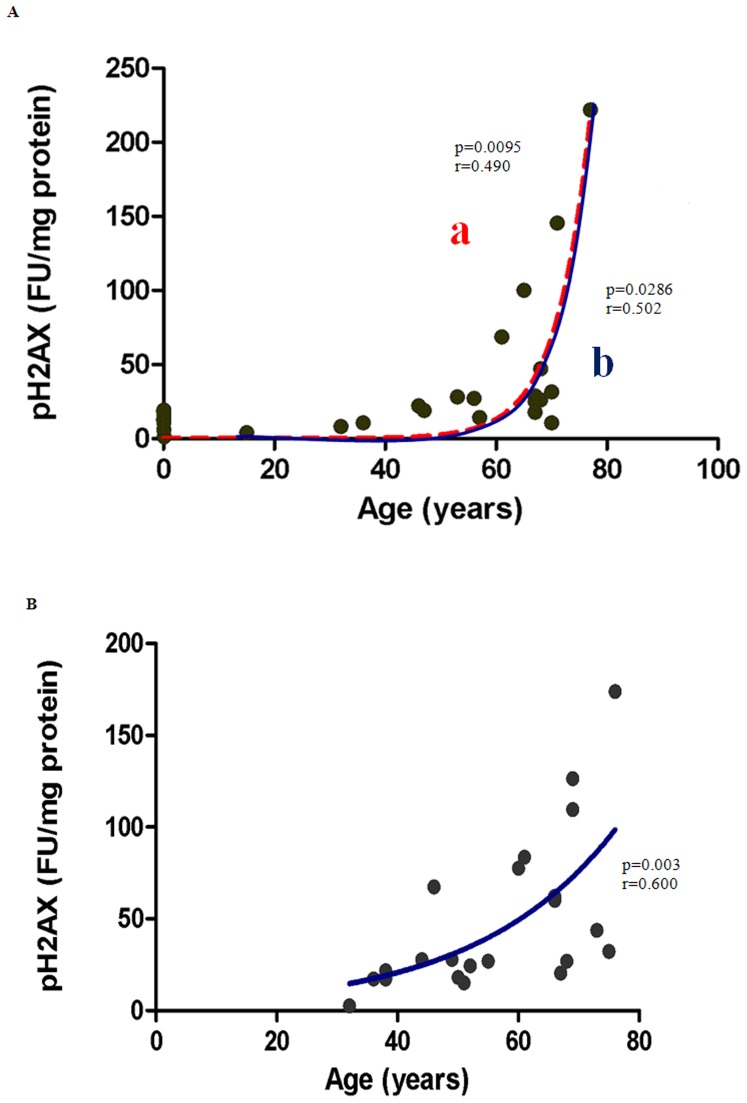
Correlation between Phosphorylation of H2AX at Ser139 and age in both (A) Males (B) Females. (**A**) P**hosphorylated-H2AX as a marker for DNA damage in male subjects show a significant correlation with age.** Line a represents the correlation for subjects aged between 0–77 years (p = 0.0095; n = 27). Line b shows post-pubescent data (males only) (p = 0.0286; n = 19). Pearson's Correlation coefficient for normally distributed data was r = 0.490 and r = 0.502 for line a, and line b respectively. An exponential (first-order) least squares fit was used to generate the nonlinear trend lines (line a and b). (**B**) **Phosphorylated-H2AX as a marker for DNA damage in post-pubescent female subjects shows a significant positive correlation with age (36–76 years) (p = 0.003; n = 22).** Pearson's Correlation coefficient for a normally distributed population was r = 0.600. An exponential (first-order) least squares fit was used to generate the nonlinear trend line.

Age-related elevation in oxidative stress can affect accumulation of DNA damage markers. DNA damage was assessed by measuring the phosphorylation of H2AX at Serine 139 using a well established fluorometric assay on isolated nuclei. DNA damage was significantly correlated with age (0–77 years) in males (Fig. 2A, line a; p = 0.0095). After excluding newborns to produce an aged matched data set comparable to our female cohort, males (Fig. 2A, line b) and females (Fig. 2B) (p<0.05) showed a similar correlation of hyperphosphorylation of H2AX with age (r = 0.502, 0.600 respectively).

### Hyperactivation of PARP occurs in Response to Oxidative DNA Damage: A Comparison between Genders

**Figure 3:**
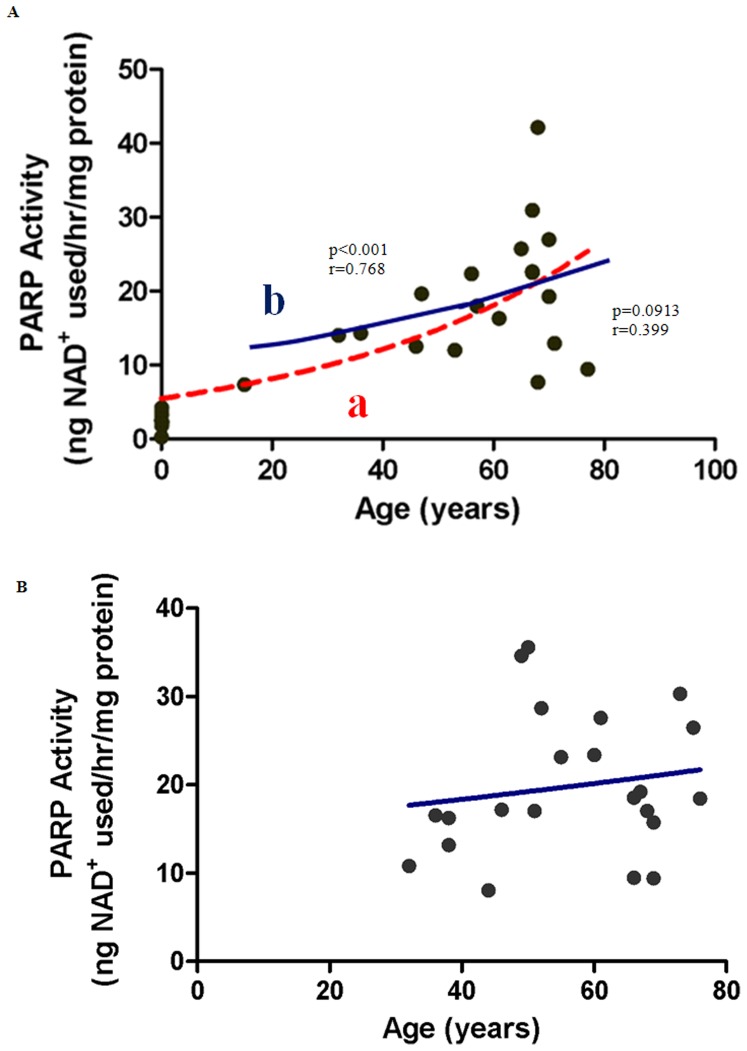
Correlation between PARP activity and aging in (A) Males (B) Females. (**A**) **PARP activity increases with age in male subjects.** PARP activity increases significantly in male subjects aged between 0–77 years (line a; p<0.0001; n = 27). The data including the post-pubescent subjects (males only) shows no significant change in PARP activity with age (line b; p = 0.0913; n = 19). Pearson's correlation coefficient was a normally distributed population was r = 0.768 and r = 0.399 for line a, and line b respectively. An exponential (first-order) least squares fit was used to generate the nonlinear trend lines (line a and b). (**B**) **PARP activity with age in female subjects (n = 27).** The apparent increase in PARP activity with age (36–76 years) is not statistically significant in post-pubescent female subjects (p = 0.4390; n = 22). Spearman's Correlation coefficient for a non-normally distributed population was r = 0.174. An exponential (first-order) least squares fit was used to generate the nonlinear trend line.

To investigate a possible association between increased oxidative stress, DNA damage and poly-ADP-ribosylation, we quantified PARP activity across the range of aging human tissue. PARP activity correlated significantly with age in males aged between 0–77 years (Fig. 3A, line a) (p<0.0001; r = 0.768). However, after newborns were excluded, no significant correlation between age and PARP activity was observed in males (Fig. 3A, line b). PARP activity and age were also not correlated in adult females, 36–75 years (Fig. 3B).

### Gender-related differences in NAD^+^ levels with Aging

**Figure 4:**
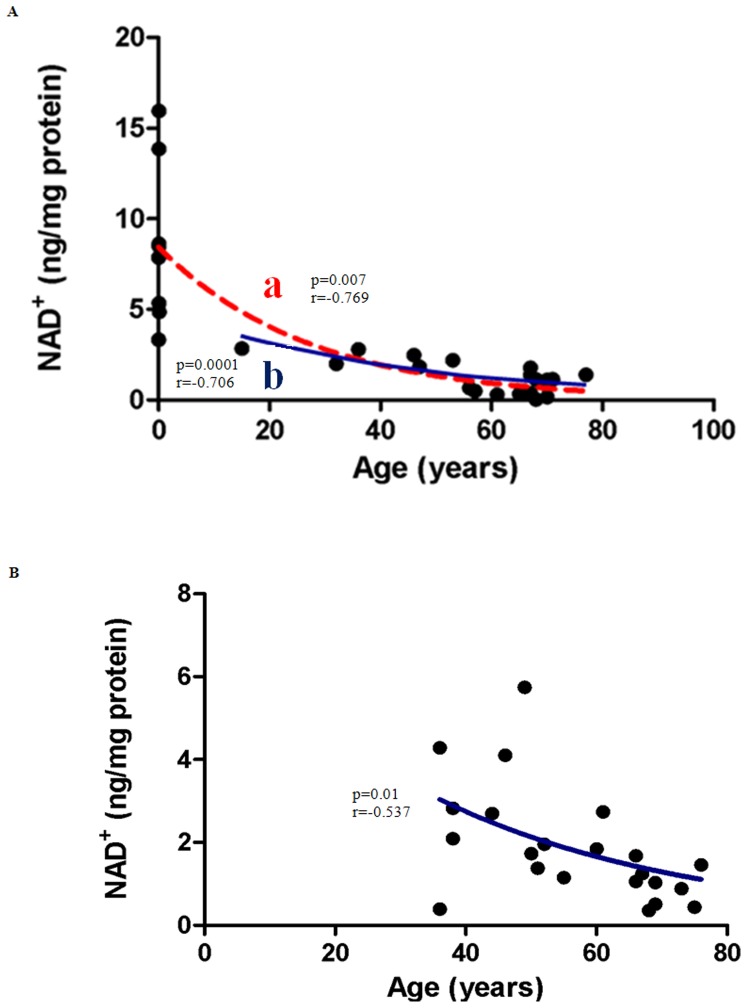
Correlation between NAD^+^ levels and Age in (A) Males (B) Females. (**A**) **NAD^+^ concentrations decline with age in males.** NAD^+^ levels decreased significantly in males aged between 0–77 years (line a; p = 0.0007; n = 27). Pearson's correlation coefficient for a normally distributed population, r = −0.769. The post-pubescent data for male subjects also showed a decline in NAD^+^ levels with age (line b; r = −0.706; p = 0.0001; n = 19). An exponential (first-order) least squares fit was used to generate the nonlinear trend lines (line a and b). (**B**) **NAD^+^ concentrations decreased significantly with age (36–76) in post-pubescent females (p = 0.01; n = 22).** Pearson's correlation coefficient for a normally distributed population,r = −0.537. An exponential (first-order) least squares fit was used to generate the nonlinear trend line.

As NAD^+^ represents the sole substrate for PARP, we quantified the effect of aging on tissue NAD^+^ levels in all specimens. NAD^+^ negatively correlated with increasing age for males aged 0–77 years (Fig. 4A line a; p<0.0007). NAD^+^ levels in post pubescent males (i.e. exclusion of newborns) (Fig. 4A, line b) and females (Fig. 4B) negatively correlated with age in both genders (r = 0.769 males, versus r = 0.537 females), p<0.05 for each.

### Age- and gender-related changes in SIRT1 deacetylase activity

**Figure 5:**
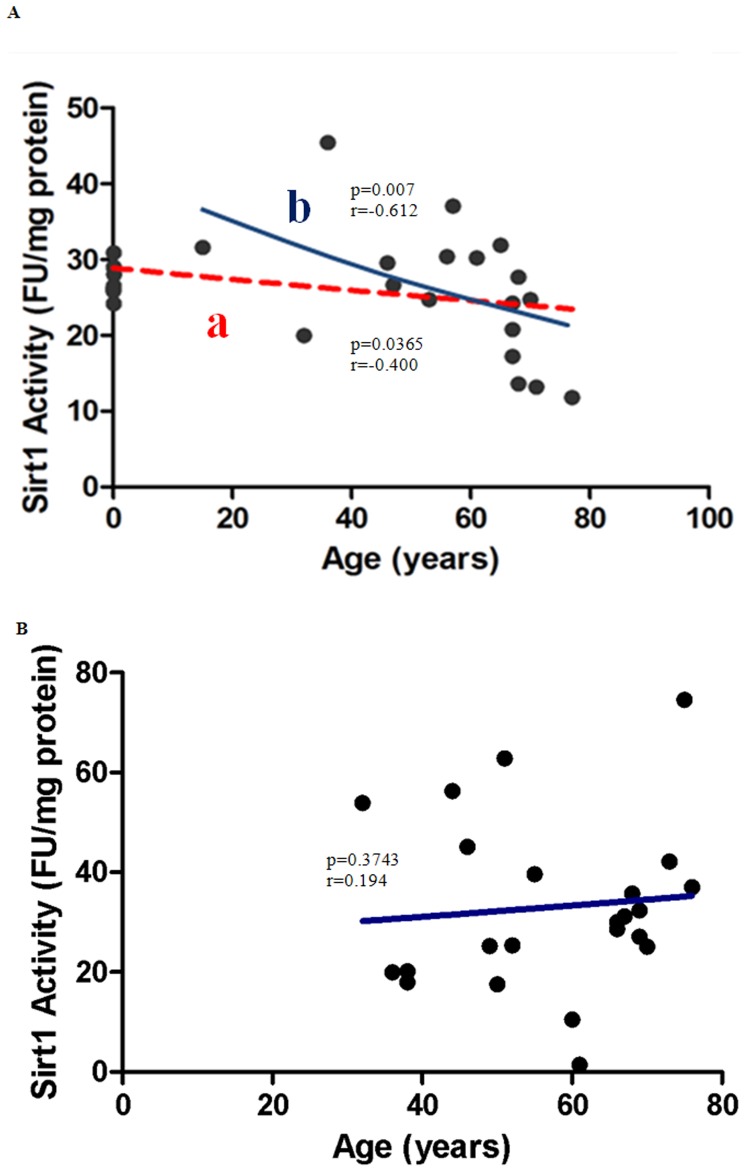
Correlation between SIRT1 Activity and Age in (A) Males (B) Females. (**A**) **SIRT1 activity declines with age in post-pubescent males.** The apparent negative correlation in SIRT1 activity with age in males aged between 0–77 years was not statistically significant (line a; p = 0.0385; n = 27). Spearman's correlation coefficient for a non-normally distributed population, r = −0.400. Line b represents the post-pubescent data shows a significant negative correlation with age (r = −0.612; p = 0.007; n = 19). An exponential (first-order) least squares fit was used to generate the nonlinear trend lines (line a and b). (**B**) **Changes in SIRT1 with age in females.** The apparent increase in PARP activity with age (36–76 years) is not statistically significant (p = 0.3743; n = 22). Spearman's correlation coefficient for a non-normally distributed population,r = 0.194. An exponential (first-order) least squares fit was used to generate the nonlinear trend line.

SIRT1 activity was investigated to assess the potential effect of age on this NAD-dependent histone deacetylase enzyme. We observed a significant negative correlation between age and SIRT1 activity for males aged between 0–77 year olds (Fig. 5A; line a; p = 0.0385). The significance was further enhanced when the newborn tissue data was excluded to allow an age-matched comparison with females (Fig. 5A, line b; p = 0.0070). Unexpectedly, no significant age related effects on SIRT1 activity were observed in females (Fig. 5B; p = 0.1943).

### Correlation between related biochemical entities (DNA damage, PARP and NAD^+^)

**Figure 6:**
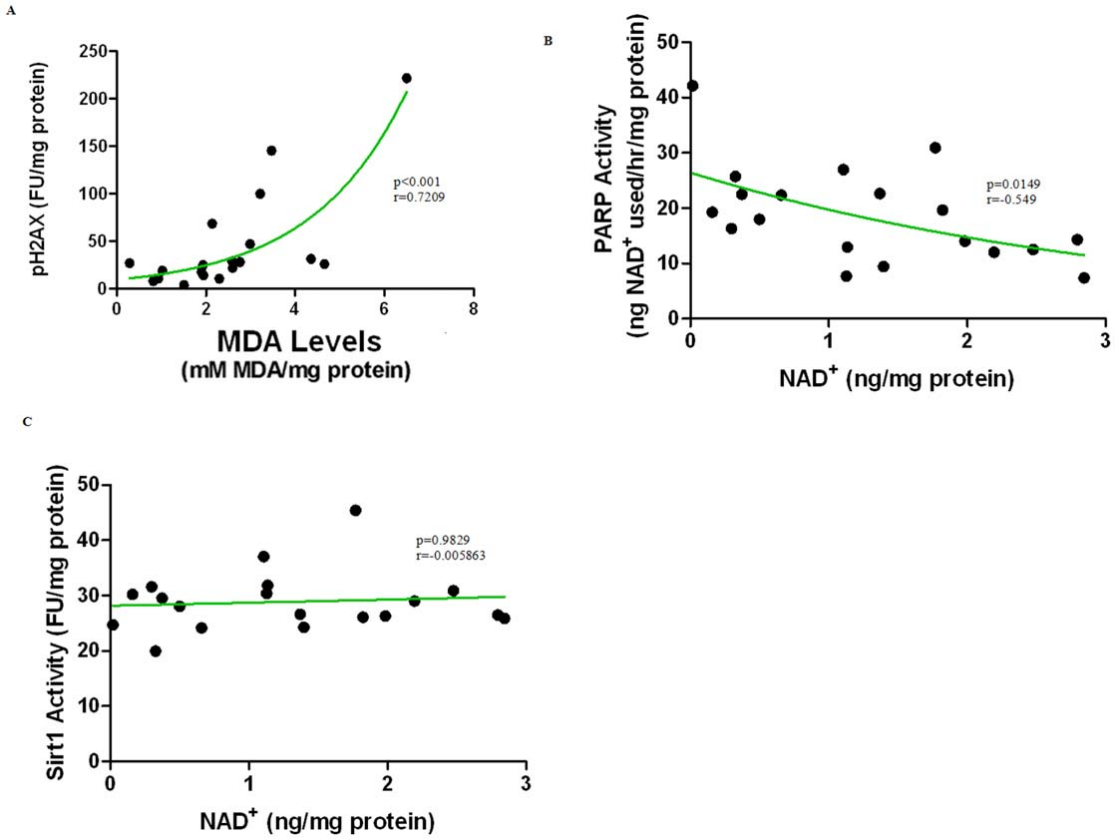
(**A**) **Lipid peroxidation increases significantly in parallel to increased DNA damage in post pubescent males (p<0.0001).** The subjects were aged between 15–77 years (n = 19). Spearman's correlation coefficient, r = 0.7209. An exponential (first-order) least squares fit was used to generate the nonlinear trend line. (**B**) **PARP activity decreases in line with a decline in NAD^+^ levels in post pubescent male subjects (p = 0.0149). .** The subjects were aged between 15–77 years (n = 19). Pearson's correlation coefficient,r = −0.5491 An exponential (first-order) least squares fit was used to generate the nonlinear trend line. (**C**) **Correlation between NAD^+^ levels and SIRT1 activity in post pubescent male subjects.** The subjects were aged between 15–77 years (n = 19). The correlation is not of statistical significance (p = 0.9829). Pearson's correlation coefficient,r = −0.005263.

We found a significant positive correlation between lipid peroxidation and DNA damage (Fig. 6A; r = 0.642; p = 0.0003), and DNA damage and PARP activity (Fig. 6D, r = 0.391; p = 0.0439) in the male cohort. As would be predicted, a highly significant negative correlation was also observed between PARP activity and NAD^+^ levels (Fig. 6B, r = −0.638; p = 0.0003). Unexpectedly however, we observed no significant correlation between NAD^+^ levels and SIRT1 activity (Fig. 6C, r = 0.059; p = 0.7715) in the male cohort and no significant correlations between any of these same parameters in the female cohort (data not shown).

## Discussion

### Lipid Peroxidation Increases with Age

There is now considerable evidence that oxidative damaged cellular structures play key roles in cellular degeneration and premature senescence. Accumulation of the lipid peroxide, MDA is one of the earliest markers of oxidative stress in animals and humans. These reactive substances can cause damage to cellular membranes and other integral cellular components [42]. Previous work by our laboratory showed an age-related increase in lipid peroxidation in a number of organ systems in physiologically aged female Wistar rats [36]. In the present study, we compared differences in lipid peroxidation levels in human pelvic skin tissue across a wide age range; from the very young to the elderly. While our results did show a clear increase in lipid peroxidation with age in human tissue in the whole cohort (Table 1), there were gender differences. MDA formation had a significant positive correlation with age in Males (Fig. 1A, line a) but was less obvious in females (Fig. 1B). Previous studies have reported increases in plasma peroxidation levels in older humans [43], [44]. Two previous studies have also reported lower plasma MDA and 8-isoprostaglandin F2a levels in healthy females compared to age-matched males [45], [46]. Measurements of basal MDA production in the aorta of female rats have also been shown to be significantly lower than that of age matched male rats [47]. The increased lipid oxidation in human tissue in males suggests an increase in oxidative potential which may be linked to either greater muscle mass and therefore, increased oxygen respiration in males and/or differences in ‘hormone related’ antioxidant capacity. However, identification of the specific contributing factors requires further investigation.

### Oxidative DNA Damage Accumulates with Age

DNA is also vulnerable to oxidative damage which, if not repaired, can trigger mutagenesis and/or cell death via energy restriction. Different markers for assessing DNA damage have been used in different studies, with 8-OHdG being the most commonly reported. However, phosphorylated H2AX is arguably a more specific reporter of general DNA damage [48] and was therefore used in this study.

Our results showed a highly significant increase in phosphorylated H2AX for both males and females with increasing age (Table 1, Fig. 2A, B). This is consistent with previous results from animal studies in our laboratory [36]. Other studies using 8-OHdG have also observed age associated increases in 8-OHdG in both rats [49], and humans [50].

While the increase in DNA damage was significant in both males and females, gender differences were apparent and have been reported in the literature. Ryberg et al. (2004) observed that DNA damage in normal lung tissue was greater in females than in males [51]. On the other hand, another study reported that DNA damage is higher in males than females in an age-matched Indian population [52]. Adding to the confusion, the observed increase in DNA damage with age in our study correlated strongly with increased lipid peroxidation in males (Fig. 6A) but not in females. As variation in age, lifestyle, occupation, and environmental exposure between individuals in the study cohort are likely to influence the levels of lipid and DNA damage future studies controlling for these various factors may be required to clarify these discrepancies.

### PARP Activity increases with age leading to NAD^+^ Depletion

It is well established, that oxidative DNA damage activates the NAD-dependent DNA repair enzyme, PARP, which is involved in base excision repair [22]. We have previously shown that PARP activity was higher in various tissues in old (24 month) compared to young (3 month) animals [36]. Messripour et al. (1994) showed that PARP activity increased in rat glial and neuronal cell cultures derived from 30 month old rats compared to 3 month old controls [53]. Grube and Burkle (1992) reported that PARP activity was increased in mammalian leukocytes. Similarly, Muiras et al. (1998) reported increased PARP activity in cells derived from centenarians. The observed increase in PARP activity is significant given the importance of PARP activation in the pathogenesis of age-related disease. Love et al. 1999, reported increased ADP ribosylation in the temporal and frontal cortex in the brains of Alzheimer's patients compared to controls [54]. Other investigators have linked PARP1 hyperactivity to degenerative diseases such as diabetes, 1-methyl-4-phenyl-1,2,3,6-tetrahydropyridine (MPTP)-induced parkinsonism and injury induced brain disorders [1], [22], [55]–[58].

Although low to moderate PARP activity plays a restorative role following genotoxic insult [59], increased PARP activity may deplete cellular NAD^+^ concentrations. We investigated whether NAD^+^ levels correlated with age and/or PARP activity in human tissue, and report for the first time that PARP activity increases with age in human skin (Table 1) and correlates with both age (Fig. 3A) and NAD^+^ depletion (Fig. 6B) (in males). Other investigators have also recently reported that PARP activity increases in mononuclear cells obtained from older males but not in females, again supporting a potential hormonal regulation of processes influencing PARP activity [60]. Further investigation using a larger cohort is likely required to clarify these apparent gender differences.

In addition, this study is the first to show that NAD^+^ levels decrease with age in human tissue. This is important since NAD^+^ is required for a number of important cellular processes including, ATP production, as cofactor for a range of enzymes, DNA repair (through PARP activity) and nuclear signaling (through sirtuin activity). Critical depletion of NAD^+^ results in cell death through reduced ATP production and activation of apoptosis [61]. Maintenance of intracellular NAD^+^ may therefore be beneficial in counteracting at least some of the age-related cellular degenerative processes.

### SIRT1 deacetylase activity in human aging

Apart from its role in genomic DNA repair, NAD^+^ also serves as the sole substrate for the sirtuin enzymes, which are silent information regulators of gene transcription. In the last decade, numerous studies have shown that gene silencing by this class of enzymes is associated with cellular resistance to genotoxic insult [62]. SIRT1 regulates the acetylation status of several metabolic transcription factors, including the peroxisome proliferator-activated receptor-γ (PPARγ), tumour suppressor protein (p53), and the FOXO forkhead family of transcription factors [63]. Over-expression of SIRT1 has a myriad of beneficial effects in lower order organisms and mice [32]. Bordone and colleagues (2007) showed that these transgenic mice share phenotypes with close similarity to mice on a calorie-restricted diet, including lower body weight, higher metabolic activity, reduced blood cholesterol levels, insulin and fasted glucose. Moreover, these rodent models were more glucose tolerant than the wild-type controls [32]. Increased SIRT1 activity has therefore been proposed as a pharmacological target for the treatment of various degenerative diseases, in particular the age-related vascular, respiratory, hepatic, and renal disorders. However, it should be remembered that SIRT1 activity is dependent on adequate NAD^+^ availability. Recently, our laboratory reported a significant decline in SIRT1 activity consistent with a decline in NAD^+^ levels in the heart, lung, liver and kidney in an aging rat model [36]. This was consistent with the hypothesis that NAD^+^ levels impact on SIRT1 activity [33].

While several studies have demonstrated the protective role of naturally occurring putative sirtuin activators such as resveratrol during aging, limited information is available regarding changes in SIRT1 deacetylase activity with age or changing NAD^+^ levels, particularly in human tissue. We therefore investigated the possible relationship between changing NAD^+^ levels with age and SIRT1 activity in human non-sun exposed pelvic skin. Given the dependence of SIRT1 activity on NAD^+^ availability, it seems logical that PARP-mediated NAD^+^ depletion would lead to a reduction in SIRT1 activity during aging. Consistent with this prediction we report for the first time a significant decline in SIRT1 activity with age in post pubescent males (Fig. 5A, line b), though surprisingly, not in females (Fig. 5B). We also did not find a correlation between NAD^+^ levels and SIRT1 activity in males (Fig. 6C). While additional studies are needed to establish the consistency of these observations, two possible explanations are suggested: 1) the gender discrepancies in declining SIRT1 activity with age may be linked to differences in the SIRT-1 promotion of androgen/oestrogen function [64]; and/or 2) the lack of correlation between NAD^+^ levels and SIRT1 activity suggests that NAD^+^ availability, though required, is not the most sensitive modulator of SIRT1 activity in humans.

## Conclusion

An extensive body of evidence has demonstrated the importance of oxidative stress in its contribution to the aging process. This study reports for the first time a link between oxidative stress and PARP activity, aging and a decline in NAD^+^ levels, in human tissue. The observed correlation between NAD^+^ levels and aging adds weight to the idea that NAD^+^ may play a role in cell senescence and longevity and not simply as an electron carrier. Overall, the results of this study suggests that our original hypothesis that oxidative stress increases with age in human skin, leading to a PARP mediated decline in NAD^+^ levels can only be reached in male humans. Despite this, some uncertainties still exist, in particular, the difference observed between our male and female cohorts and of the link between the age associated decline in NAD^+^ levels and SIRT1 activity. While further research with larger numbers may yet demonstrate PARP and/or SIRT1 correlation with reduced NAD in females another explanation may be that females have a greater capacity to recycle NAD^+^ from the PARP metabolite nicotinamide (e.g. through increased NAMPT activity). Further studies are required to test this hypothesis. However, the current study does provide credible data supporting the importance of maintaining NAD^+^ levels, with age for maintaining PARP activity. While it appears logical that this negative OS/age-induced biochemical cascade may be achieved by increasing NAD^+^ stores and thereby enhancing PARP and SIRT1 activities, the therapeutic benefit of this approach in humans is yet to be established.
